# Life cycle assessment and sensitivity analysis of carbon emissions in full depth reclamation with portland cement and conventional pavement repair

**DOI:** 10.1038/s41598-025-03635-y

**Published:** 2025-05-28

**Authors:** Haiwei Zhang, Qingqing Zhang, Chuangdan Luo, Ning Liu, Ke Wang

**Affiliations:** 1 School of Civil and Environmental Engineering, Zhengzhou University of Aeronautics, Zhengzhou, 450046 People’s Republic of China; 2 Henan Provincial Engineering Laboratory of High Permeability Pavement Materials, Zhengzhou, Henan, 450046 China; 3 Henan Provincial Engineering Technology Research Center of Modified Asphalt Pavement Materials, Zhengzhou, Henan, 450046 China; 4 Henan Zhongping Jiaoke Research and Design Institute Co., Ltd, Pingdingshan, 467000 People’s Republic of China

**Keywords:** Pavement rehabilitation, Full-depth reclamation with Portland cement, 3D-Move, Pavement life prediction, Carbon emission calculation, Sensitivity analysis, Civil engineering, Materials science

## Abstract

Full-depth reclamation with Portland cement (FDR-PC) is a pavement rehabilitation technology that has garnered significant attention and research interest due to its ability to fully utilize existing pavement materials in situ and address deep structural issues within the pavement. This paper evaluates the advantages of FDR-PC in terms of carbon emission compared to traditional asphalt pavement rehabilitation technologies. Firstly, under the same service life conditions, the structural configurations of asphalt pavements were designed using 3D-Move Analysis for three different technologies: removal and reconstruction, cold central plant recycling, and FDR-PC. Subsequently, carbon emission models were established based on the life cycle assessment (LCA) method and the construction processes, allowing for a comparison of carbon emissions and energy consumption among the three technologies. Finally, a sensitivity analysis was conducted to assess the impact of various factors on carbon emissions during the FDR-PC construction process. The results indicate that in terms of carbon emissions from the pavement base layer, FDR-PC accounts for 92% and 90% of those produced by removal and reconstruction and cold central plant recycling, respectively, while its energy consumption is 60% and 70% of the latter two technologies. Notably, during the transportation phase, FDR-PC demonstrates carbon emissions and energy consumption levels at merely 4% each compared to conventional removal and reconstruction, and 6% each relative to cold central plant recycling. The sensitivity analysis further reveals that the cement content is the most influential factor affecting the carbon emissions of FDR-PC.

## Introduction

As of 2023, the total mileage of China’s ordinary highways has reached 5.25 million kilometers, with asphalt pavement serving as the predominant paving technique. However, over time and with increasing traffic loads, asphalt pavements are prone to structural deterioration, including rutting, cracking, and potholes.

Several widely utilized pavement rehabilitation technologies are available to address the rehabilitation needs of China’s ordinary highways. The first is the removal and reconstruction, which involves completely excavating the old pavement and laying new materials. While this approach is costly and time-intensive, it offers reliable results. The second category includes recycling techniques, such as cold central plant recycling, which reclaims old asphalt materials and stabilizes them with additives at a factory before repaving. This method conserves resources and reduces environmental impacts. In recent years, an innovative recycling technology, known as FDR-PC, has gained widespread adoption. This method employs specialized equipment to crush and regenerate both the surface and base layers of aged pavements directly on-site. By minimizing construction time and enhancing rehabilitation efficiency, FDR-PC is particularly well-suited for large-scale segments suffering from severe structural damage.

Current research on FDR-PC primarily focuses on the performance of the mixture, with design guidelines from various countries predominantly utilizing the 7-day unconfined compressive strength as a key design parameter^1^. Laboratory studies have extensively investigated the influence of the primary components of FDR-PC on its strength. The incorporation of reclaimed asphalt pavement (RAP) into new road materials facilitates the recycling of old pavement waste, thereby conserving resources. However, RAP has been found to accelerate the initiation and propagation of cracks within the mixture. Numerous studies have demonstrated that higher RAP content reduces the strength and stiffness of the mixture^2–4^ and diminishes its fatigue life^5–7^. In contrast, cement exhibits the opposite effect. Multiple studies indicate that increasing the cement content enhances the strength and stiffness of the mixture^2,8^, as well as its durability^5,9,10^. Nevertheless, due to the shrinkage effects associated with cement, its content is typically limited to no more than 6%^10^. Furthermore, the compaction method significantly influences the density of the mixture, thereby affecting its porosity and, ultimately, its strength and stiffness^2,6,10^. Additionally, the curing duration and temperature play a critical role in the curing process, impacting the mixture’s strength and stiffness through shrinkage effects^2,6,11^. Similarly, field studies have primarily concentrated on the long-term stability and service life of FDR-PC pavements^12,13^.

Against the backdrop of China’s *Dual Carbon* policy framework, China has established ambitious targets to achieve peak carbon emissions by 2030 and carbon neutrality by 2060. The Chinese government has introduced the *1 + N* policy system to propel energy transition and advance green, low-carbon technologies, aiming to reduce the carbon footprint in sectors such as transportation and construction. However, research on carbon emissions associated with FDR-PC remains relatively limited^1^. Current studies on FDR-PC predominantly focus on its structural performance and economic benefits, while its potential for carbon reduction and life-cycle carbon footprint has not been thoroughly investigated. In light of China’s vigorous pursuit of its *Dual Carbon* objectives, it is imperative to intensify research on the carbon emissions of FDR-PC. Such efforts will facilitate a more comprehensive evaluation of its contributions to low-carbon development in the highway sector.

The methodologies for calculating carbon emissions include direct measurement, process-based LCA, input-output-based LCA, and hybrid LCA^14–16^. Among these, the process-based LCA quantifies the consumption and emissions of each discrete process within the life cycle system, aggregating data from individual processes to compute the overall inputs and emissions across the entire life cycle. This method, which can be represented using flowcharts to facilitate independent analysis, is widely employed and has been standardized by the International Organization for Standardization (ISO)^17,18^. It calculates carbon emissions by multiplying activity data by corresponding emission factors, thereby establishing a formula for carbon emission estimation. This approach is extensively utilized in environmental research and carbon footprint assessments due to its ability to provide rapid estimates of carbon emissions without the need for complex models.

Suprayoga et al.^19^ conducted a comprehensive analysis of 31 scholarly articles to evaluate the extent to which sustainability assessments have been applied in road infrastructure projects, focusing on the environmental, economic, and social pillars. Their findings revealed that the *project evaluation* approach encompasses the broadest range of criteria and is recommended as the most suitable decision-making methodology.

Del Ponte et al.^20^ employed the LCA method to analyze nine highway projects in the United States, calculating the energy and water consumption associated with the use of recycled materials. This study also assessed the economic cost savings and environmental impacts resulting from the utilization of recycled materials. Vidal et al.^21^ utilized the LCA method to compare the carbon emissions of hot-mix asphalt mixtures and zeolite-based warm-mix asphalt mixtures. Chen et al.^22^ based on an airport runway rehabilitation project, examined the environmental benefits of RAP under various scenarios. The study analyzed energy consumption and carbon emissions during pavement construction, taking into account factors such as RAP content, water content, and blending efficiency. Liu et al.^23^ investigated 20 asphalt road projects and 18 concrete road projects in China. The results indicated that the carbon emissions per kilometer per lane were approximately 500 tons for concrete roads and 1,250 tons for asphalt roads.

Several studies have conducted comparative analyses of the environmental impacts associated with FDR and conventional reconstruction. Amarh et al.^24^ evaluated the carbon emissions of various stabilization techniques—including lime, cement, foamed asphalt, and emulsified asphalt—applied in FDR, as well as those of cold in-place recycling and non-recycling methods, based on multiple technical scenarios for pavement projects in Virginia. Their findings demonstrate that recycling technologies exhibit a lower global warming (GW) compared to non-recycling technologies during the reconstruction phase. Over the entire lifecycle, approximately 98% of GW emissions originate from pavement-vehicle interaction during the use phase, underscoring the critical importance of optimizing initial pavement smoothness and reducing annual deterioration rates to mitigate emissions. Souza et al.^25^ performed a quantitative assessment of carbon emissions and energy consumption associated with FDR and Mill and Fill, revealing that FDR offers superior environmental benefits. By eliminating the need for asphalt heating, FDR reduces greenhouse gas emissions by 51% and energy consumption by 64%. Schmitt et al.^26^ further compared the environmental performance of stabilized FDR, non-stabilized FDR, and reconstruction. Their results indicate that stabilized FDR outperforms both non-stabilized FDR and reconstruction in environmental metrics. The study highlights that reconstruction performs poorest across most environmental indicators, while the emission reduction efficacy of stabilized FDR is significantly influenced by transportation distance and design lifespan. Most of the reviewed studies, when analyzing and comparing the environmental impacts of different technologies, either set the functional unit to identical road parameters or merely evaluated the carbon emission per unit mass of material, without considering the differences in road performance. Consequently, this paper aims to assess the environmental impacts of three typical technologies based on the same road service life.

**Fig. 1:**
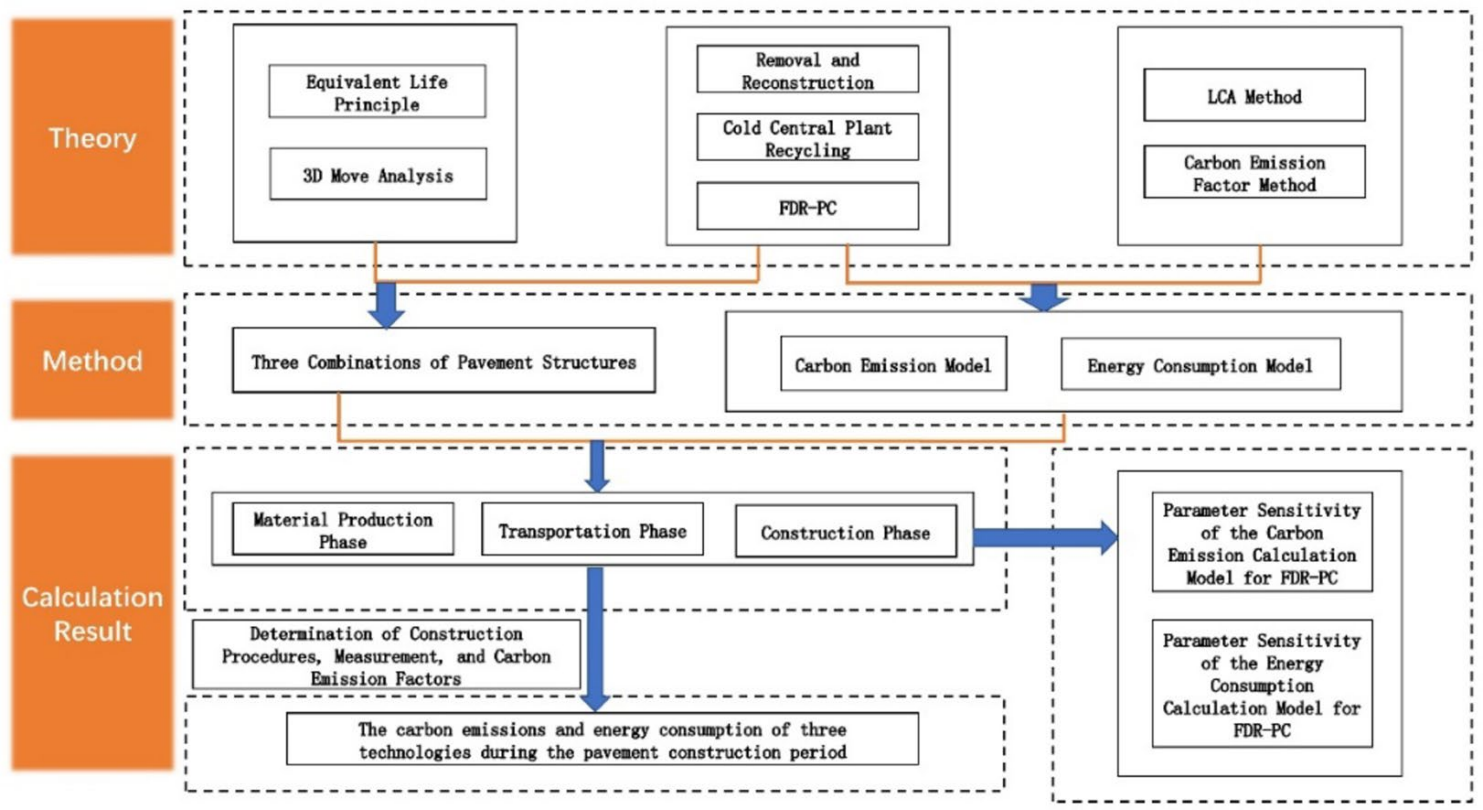
The technical roadmap of this paper.

This study is grounded in a rehabilitation project of ordinary asphalt pavement on a Chinese highway, evaluating the carbon emissions of three major rehabilitation technologies under the criterion of identical pavement service life. Initially, the 3D Move Analysis software was employed to calculate the pavement structures corresponding to the three technologies, ensuring consistency in service life, and thereby establishing the technical frameworks. Subsequently, based on the LCA method, the highway rehabilitation process was segmented into three distinct phases: the material production phase, the transportation phase, and the construction phase. Utilizing the carbon emission factor method, a formula for calculating carbon emission was developed, and the actual project data were applied to quantify the carbon emissions for each of the three technologies. Finally, a sensitivity analysis was conducted on the parameters within the carbon emission formula for the FDR-PC technology. The findings of this research aim to provide a foundational basis for the advancement and optimization of FDR-PC technology, contributing to more sustainable practices in pavement rehabilitation. The technical roadmap of this paper is listed in Fig. 1.

## Pavement structure design

### Pavement design index

Given the diversity of pavement structure forms and the complexity of performance influencing factors, the *Design Specification for Highway Asphalt Pavements*^27^ in China has established five performance analysis models for pavement damage, namely, low-temperature cracking of pavement, fatigue cracking of asphalt layer, permanent deformation of the asphalt layer, fatigue cracking of inorganic binder base, and permanent deformation of subgrade. The parameters of these five models are the bottom tensile strain of the asphalt overlay layer, the low-temperature shrinkage cracking index of the asphalt layer, the permanent deformation amount of the asphalt overlay layer, the vertical compressive strain at the top of the residual layer (during road renovation or reconstruction, the original pavement structure might not be entirely demolished. Instead, a portion of it is left behind and incorporated as part of the new pavement structure. This remaining part is what we term the “residual layer”), and the fatigue life of the inorganic binder. When determining the pavement structure performance, the calculation parameters need to be selected from these parameters.

Firstly, regarding the bottom tensile strain of the asphalt overlay layer, the value for FDR-PC pavement is consistently below 70µε^28–30^. Hence, this parameter can be disregarded. Concerning the low-temperature shrinkage cracking index of the asphalt surface layer, the specification^27^ indicates that it is only related to the temperature characteristics of the pavement application area and the type of subgrade soil, and has no relation to the pavement structure characteristics. For the permanent deformation of the asphalt overlay layer, the specification^27^ suggests that provided the asphalt mixture satisfies the rutting test requirements, this parameter may not be considered as a design index for FDR-PC pavements. Regarding the vertical compressive strain at the top surface of the residual layer, the specification^27^ states that when the calculated modulus of the recycled layer reaches 4000 MPa or above, it is no longer considered as a pavement design index. Therefore, only the fatigue cracking index of the inorganic binder-stabilized base course is related to the structural performance of the FDR-PC pavement.

The fatigue model for the inorganic binder-stabilized layer, as specified in the specification^27^, is presented in the formula (1).

1 $$\:{lg}{N}_{e2}=a-b\left(\frac{{\sigma\:}_{t}}{{R}_{s}}\right)+{k}_{c}-0.57\beta\:+{lg}(\frac{{k}_{a}}{{k}_{T2}})$$

*Ne*_*2*_ represents the fatigue life of the inorganic binder layer along the axis; *σ*_*t*_ denotes the bottom tensile stress (MPa) of the inorganic binder stabilization layer; R_s_ stands for the flexural and tensile strength (MPa) of inorganic binder stabilized materials; k_T2_ is a temperature adjustment coefficient, determined as 1.30 according to Appendix G of the specification^27^; k_a_ is an adjustment coefficient for seasonal frozen soil areas, determined as 1.00 according to the specification^27^; a and b are regression parameters for fatigue testing; k_c_ is a field comprehensive correction factor, determined according to formula (2).

2 $$\:{\text{k}}_{\text{c}}={\text{c}}_{1}{\text{e}}^{{\text{c}}_{2}({\text{h}}_{\text{a}}+{\text{h}}_{\text{b}})}+{\text{c}}_{3}$$

In the newly constructed road surface layer, the values of $\:{c}_{1},\:{c}_{2}$, and $\:{c}_{3}$ are 14, -0.0076, and − 1.47, respectively. For the rehabilitated road base layer, these parameters are 18.5, -0.01, and − 1.32, respectively. $\:{h}_{a}$ and $\:{h}_{b}$ represent the thicknesses of the asphalt mixture layer and the inorganic binder-stabilized layer above the calculation point, respectively. β denotes the target reliability index, with a value of 1.04 for secondary highways and 0.84 for tertiary highways.

The predicted fatigue life $\:{N}_{e}$ must exceed the cumulative equivalent single-axle load applications over the design period for the designated traffic lane. Based on this criterion, the range of values for the pavement structure can be derived.

### Pavement structure model

According to the standard, the moduli of the newly built (new base for cement stabilized crushed stone base), cold central plant recycling, and FDR-PC regeneration bases were determined to be 8150 MPa, 7450 MPa, and 6050 MPa, respectively. Their flexural-tensile strengths were 1.3 MPa, 1.2 MPa, and 1.1 MPa, respectively, based on specimen forming, curing, resilience modulus and bending experiments, as well as the review of relevant literature. The modulus and flexural tensile strength of cold central plant recycling and FDR-PC regeneration base are lower than those of newly built ones for many reasons, including material quality, regeneration process, construction quality control, structural design, parameter selection and environmental factors. 3D-Move Analysis software regards each pavement structure layer as a continuous whole. By defining load, material and other parameters, and using Fourier transform technology, the mechanical response analysis of the pavement structure is completed, and the displacement stress-strain value of each pavement structure layer is simulated. The three-dimensional continuous finite layer method has the advantages of fast operation, less meshing and high reliability of results. As shown in Table 1, the pavement structure parameters were input by 3D-Move software. Static simulation (at 20℃ and 10 Hz) was performed on the newly built pavement structure under the conditions that the BZZ-100 single axle and double wheel set were designed for axle load, tire pressure was 0.7 MPa, load was 25Kn, load radius was 0.107 m, and center distance between two wheels was 0.321 m.

**Table 1:** New pavement structure.

Pavement structure layer	Material	Structural layer thickness	Poisson’s ratio	Modulus (Mpa)	Density (Kn/m^3^)	Damping ratio
Upper layer	AC-13	4 cm	0.25	8549	24	10%
Lower layer	AC-20	6 cm	0.25	7380	23.5	10%
Base layer	Cement stabilized crushed stone base(CSCS)	20 cm	0.25	8150	22.5	0
Residual layer (roadbed)	Graded crushed stone	0(Infinite half space body)	0.4	150	19	0

### Determination of base thickness

**Fig. 2:**
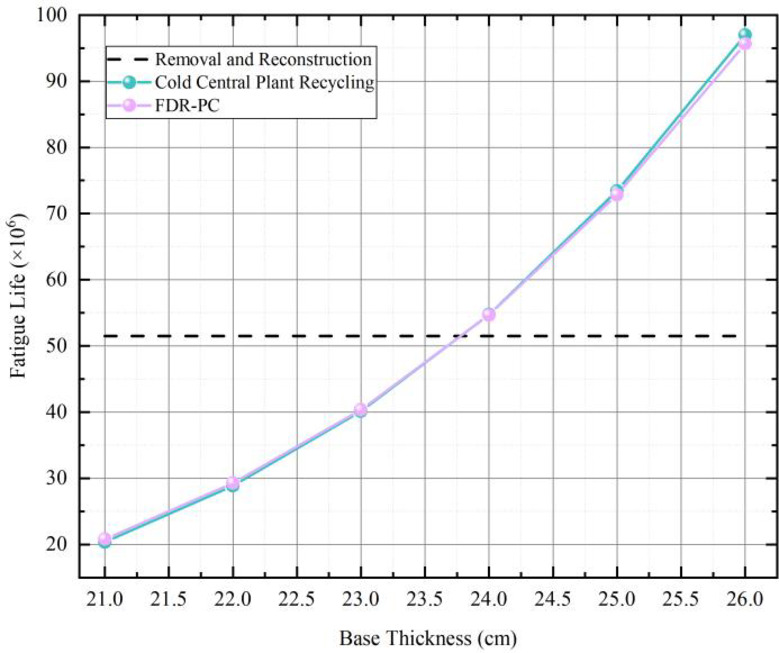
Fatigue life under different base thicknesses.

Using 3D-move software to simulate the static behavior of the newly constructed subgrade pavement, determine the bottom tensile stress of the inorganic binder stabilized layer, and by inputting the formula (1), the fatigue life of the inorganic binder layer can be calculated. The fatigue life of the newly constructed subgrade can be calculated to be approximately 5.15 × 10^6^ axle passes. Using 3D-move software to simulate the structural parameters of the cold central plant recycling and FDR-PC pavement, by adjusting the thickness of the base layer, determining the bottom tensile stress value of the base layer, and inputting the formula (1), (2) into its three types of pavement parameters. As shown in Fig. 2, after simulating and calculating the fatigue life of the cold central plant recycling and FDR-PC base thickness from 21-26 cm, it can be seen that when the fatigue life of the new base is about 51.5 × 10^6^ axis times, and the cold central plant recycling and FDR-PC base thickness is 23–24 cm and 23–24 cm, the fatigue life of the cold central plant recycling and FDR-PC base thickness is about 51.5 × 10^6^ axis times, The fatigue life of 40.1 × 10^6^-54.7 × 10^6^ axis times and 40.3 × 10^6^-54.7 × 10^6^ axis times are similar.

**Fig. 3:**
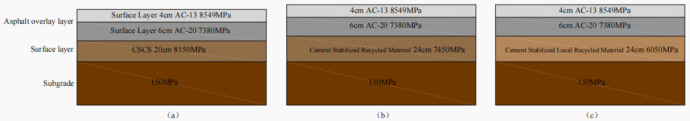
Pavement structure, (**a**) Removal and reconstruction, (**b**) Cold central plant recycling, (**c**) FDR-PC.

The base layer thickness of cold central plant recycling and FDR-PC regeneration can be selected to be 24 cm and 24 cm respectively, and the pavement structure diagram of the three processes is drawn as shown in Fig. 3. Therefore, according to the fatigue life prediction equation and mechanical response 3D-move simulation calculation, the following three pavement structures are finally recommended:

1. Removal and reconstruction: asphalt overlay layer (surface layer 4 cm, 8549 MPa + surface layer 6 cm, 7380 MPa) + base layer (20 cm CSCS, 8150 MPa) + Subgrade (150 MPa);
2. Cold central plant recycling: asphalt overlay layer (surface layer 4 cm, 8549 MPa + surface layer 6 cm, 7380 MPa) + base layer (24 cm cement stabilized recycled material, 7450 MPa) + Subgrade (150 MPa);
3. FDR-PC: Asphalt overlay layer (surface layer 4 cm, 8549 MPa + surface layer 6 cm, 7380 MPa) + base layer (24 cm cement stabilized local recycled material,6050 MPa) + Subgrade (150 MPa).

## Method

### System boundary and functional unit

This study primarily investigates the carbon dioxide emissions and energy consumption throughout the life cycle of rehabilitation of ordinary highways utilizing recycled materials. Grounded in the LCA method, the life cycle of ordinary highway rehabilitation is delineated into three distinct phases: the material production phase, the transportation phase, and the construction phase.

The research is based on a highway project in China, with a 1-kilometer segment serving as the computational unit. The selected road segment is located within the K346 + 100-K351 + 200 section in Ulanqab City, Inner Mongolia. The pavement width measures 10.50 m, while the subgrade width extends to 11.50 m. The existing road structure comprises a 5-centimeter asphalt concrete surface layer, a 20-centimeter cement-stabilized crushed stone base layer, and an 18-centimeter cement-stabilized crushed stone subbase layer.

### Life cycle inventory analysis

#### Technical solutions

The pavement structures corresponding to removal and reconstruction, cold central plant recycling, and FDR-PC, all designed to achieve the same pavement service life, are presented in Table 2. Additionally, Table 2 provides the material densities of the existing pavement layers, as well as the material densities for each layer of the pavement structures associated with the three aforementioned technologies.

**Table 2:** Surface and base layer data for three technologies.

	Removal and reconstruction	Cold central plant recycling	FDR-PC
Density of old road materials (t/m³)	2.2	2.2	2.2
Upper Layer	4 cm AC-13		
Asphalt-aggregate ratio of asphalt mixture (Upper Layer) (%)	5.2		
Lower layer	6 cm AC-20		
Asphalt-aggregate ratio of asphalt mixture (lower layer) (%)	4.5		
Asphalt mixture density (t/m^3^)	2.35		
Base thickness(cm)	20	24	24
Wet density of new base mixture (t/m^3^)	2.25	2.2	2.15
Water content (%)	5	6	6.5
Cement content (%)	5	5	5

#### FDR-PC construction process

The construction processes of removal and reconstruction, as well as cold central plant recycling, have been extensively discussed in numerous studies concerning carbon emission analyses^31–33^. This paper, however, will focus exclusively on delineating the base layer construction process of FDR-PC.

The construction sequence for FDR-PC base layer can be delineated into several pivotal steps.

**Fig. 4:**
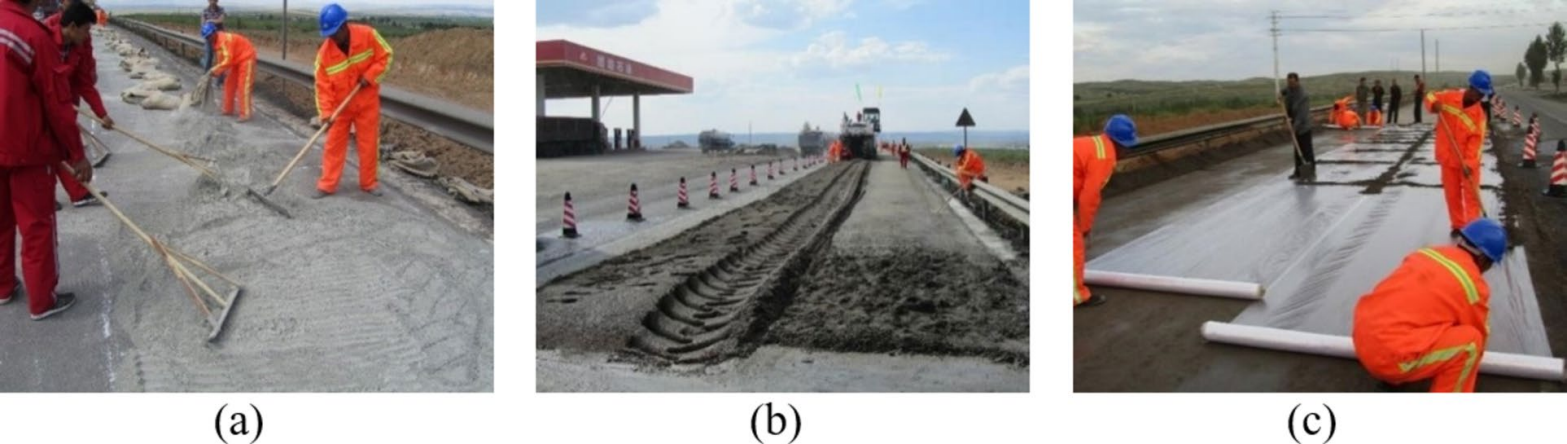
The on-site construction process of the FDR-PC, (**a**) Road closure and cement spreading, (**b**)Cold recycling, milling, and paving, (**c**) compaction and curing.

Initially, the quantity of cement required is calculated based on the thickness of the recycled layer, the maximum dry density of the mixture, the degree of compaction, the optimal water content, and the cement content. This calculated amount of cement is then uniformly distributed over the existing pavement, as illustrated in Fig. 4(a).

Subsequently, a cold recycling machine is employed to mill and crush the old pavement at a consistent speed, as depicted in Fig. 4(b). This machine thoroughly mixes the old pavement materials with cement and water, after which the recycled mixture is spread over the existing pavement.

Finally, a grader is utilized to level the recycled layer, eliminating any traces left by the recycling machine and adjusting the transverse and longitudinal gradients of the recycled layer. Once leveled, a roller is used to compact the recycled layer. Upon completion of the cold-recycled layer construction, the surface is covered with plastic sheeting for a curing period of seven days, during which vehicular traffic is strictly prohibited, as shown in Fig. 4(c).

#### Emission inventory

In the materials production phase, the surface layers of the three technologies are identical, utilizing SBS-modified asphalt and aggregates. For the base layer, removal and reconstruction employ cement, aggregates, and water, whereas cold central plant recycling and FDR-PC utilize reclaimed pavement materials, cement, and water. Carbon emissions and energy consumption in this phase arise from the energy expended during extraction, transportation, and processing, as well as the fuel consumed by machinery and equipment. Factors such as extraction methods, processing techniques, and transportation modes significantly influence the carbon emissions and energy consumption associated with raw materials.

The transportation phase encompasses three primary routes: transporting raw materials from the factory to the construction site or off-site processing facility, conveying milled old pavement materials from the construction site to the processing plant, and delivering the mixed materials from the processing plant back to the construction site. Carbon emissions during transportation predominantly stem from fuel consumption and exhaust emissions of the transport vehicles, influenced by variables such as vehicle type, transportation distance, fuel type, and the mass of the transported materials. This study directly employs actual engineering data to calculate carbon emissions and energy consumption for this phase.

During the construction phase, the surface layer construction processes are consistent across all three technologies, primarily involving the mixing and paving of asphalt mixtures. For the base layer, removal and reconstruction and cold central plant recycling involve milling the old pavement, mixing the base layer materials, paving, and compaction. In contrast, the FDR-PC entails cold recycling of the old pavement, followed by leveling and compaction of the recycled layer. Carbon emissions and energy consumption during the construction phase are attributed to the fuel consumption and exhaust emissions of the construction machinery.

#### Carbon emission factors and energy consumption factors

Table 3 presents the carbon emission factors and energy consumption factors for raw materials utilized in this study. The carbon emission and energy consumption factors for 42.5-grade cement are derived from Pan^34^, which incorporates adjustments for Chinese cement production processes and transportation based on the third-tier calculation precision provided by International Council for Environmental and Economic Development (ICEE). Data for styrene-butadiene-styrene modified asphalt (SBS-modified asphalt) are sourced from Gao^16^, which is based on the production processes of Sinopec’s SBS-modified asphalt. The data for aggregates are obtained from Gao^16^, which is grounded in the three-stage closed-circuit screening technology for aggregates.

**Table 3:** Carbon emission factors and energy consumption factors for Raw materials.

Materials	Carbon emission factor	Unit of carbon emission factor	Energy consumption factor	Unit of energy consumption factor
SBS Modified asphalt	613.00	kg/t	10576.00	MJ/t
Aggregate	3.50	kg/t	37.00	MJ/t
Cement	870.00	kg/t	3181.00	MJ/t
Water	0.20	kg/t	3.00	MJ/t

Table 4 presents the carbon emission factors and energy consumption factors for transportation machinery. The machinery involved in the transportation phase includes lorries and dump trucks. Carbon emissions and energy consumption are considered only for two stages: vehicle operation and fuel production. It is assumed that vehicles travel fully loaded to the destination and return empty, with the energy consumption of the empty return trip calculated as 70% of the fully loaded trip. Based on the vehicle’s load capacity, total mass, average operating speed, and diesel fuel consumption, the carbon emission factors and energy consumption factors are derived.

Table 4 also provides the factors for construction machinery. Utilizing fuel consumption data for machinery from the *Highway Engineering Machinery Shift Cost Quota*^35^ and carbon emission and energy consumption data for fuels from the *China Energy Statistical Yearbook*^36^, the carbon emission factors and energy consumption factors per unit mass or unit volume of material processed by the machinery can be estimated. Given that surface layer construction is a well-established technology with extensive research, this study simplifies the process by adopting a single carbon emission factor for surface layer construction, as referenced in prior studies.

**Table 4:** Carbon emission factors and energy consumption factors for machinery.

Machine and equipment	Carbon emission factor	Unit of carbon emission factor	Energy consumption factor	Unit of energy consumption factor
Lorry	0.12	kg/t·km	3.25	MJ/t·km
Dump truck	0.12	kg/t·km	4.23	MJ/t·km
Milling machine	2.53	kg/t	34.17	MJ/t
Plant mixing equipment	0.84	kg/t	4.84	MJ/t
Paver	0.27	kg/t	7.07	MJ/t
Road roller	0.58	kg/t	15.58	MJ/t
Asphalt mixture production, paving, and compaction	10.93	kg/t	263.47	MJ/t
Wirtgen cold recycler	2.62	kg/t	38.32	MJ/t
Motor grader	4.00	kg/t	0.25	MJ/t

#### Carbon emission model and energy consumption model

This paper employs CO_2_ emissions as the primary metric for calculation. In accordance with the phase delineation of the LCA method, the total carbon emission model and the total energy consumption model can be expressed as formula (3) and (4), respectively:

3 $$E={E_1}+{E_2}+{E_3}$$

4 $$m={m_1}+{m_2}+{m_3}$$

Here, $\:E$ and $\:m$ represent the total energy consumption and the total CO_2_ emissions generated during the asphalt pavement construction process, respectively. $\:{E}_{1}\:$and $\:{m}_{1}$ denote the corresponding quantities in the material production phase, $\:{E}_{2}$ and $\:{m}_{2}$ in the transportation phase, and $\:{E}_{3}$ and $\:{m}_{3}$ in the construction phase.

The calculation of carbon emissions and energy consumption in the material production phase is defined as the sum of the products of the total mass of each raw material and its respective factors, as illustrated in formulas (5) and (6):

5 $${E_1}=\sum {Q_{\text{i}}} \cdot F{C_{\text{i}}}$$

6 $${m_1}=\sum {Q_{\text{i}}} \cdot F{E_{\text{i}}}$$

$\:{Q}_{i}$ represents the mass of the raw material. $\:F{C}_{i}$ denotes the energy consumption factor per unit of raw material, measured in MJ per unit usage. $\:F{E}_{i}$ signifies the CO_2_ emission factor per unit of raw material, measured in tons per unit usage.

The carbon emissions and energy consumption during the transportation phase are defined as the product of the total workload of the transportation machinery and their respective factors. The calculation methods are presented in formulas (7) and (8):

7 $${E_2}=\mathop \sum \limits_{i} F{C_i} \cdot {n_i} \cdot {D_i}$$

8 $${m_2}=\mathop \sum \limits_{i} F{E_i} \cdot {n_i} \cdot {D_i}$$

$\:F{C}_{i}$ represents the energy consumption factor of the machinery, measured in MJ per ton-kilometer. $\:F{E}_{i}$ denotes the emission factor of the machinery, measured in kg per ton-kilometer. $\:n$ signifies the workload of the machinery. $\:{D}_{i}$ represents the transportation distance in kilometers.

The carbon emissions and energy consumption during the construction phase are defined as the product of the workload of the construction machinery and their respective factors. The specific equations are given in formulas (9) and (10):

9 $${E_3}=\sum {Q_{\text{i}}} \cdot F{C_{\text{i}}}$$

10 $${m_3}=\sum {Q_{\text{i}}} \cdot F{E_{\text{i}}}$$

$\:{Q}_{i}$ signifies the workload of construction machinery. $\:F{C}_{i}$ represents the energy consumption factor per unit workload of the machinery, measured in MJ per unit workload. $\:F{E}_{i}$ denotes the CO_2_ emission factor per unit workload of the machinery, measured in tons per unit workload.

## Result and discussion

### Calculation

#### Material production phase

The surface layers of the three technologies are identical, with the new overlay consisting of 4 cm of AC-13 and 6 cm of AC-20, utilizing medium-graded asphalt concrete. The raw materials include SBS-modified asphalt and aggregates. The carbon emissions and energy consumption of the surface layers are presented in Table 5.

**Table 5:** Energy consumption and carbon emissions of the surface layers in the material production phase for the three technologies.

Pavement layer	Material	Material quantity (t)	Unit energy consumption (MJ/t)	Unit CO_2_ emissions (kg/t)	Energy consumption (MJ)	CO_2_ emissions (kg)
Asphalt surface layer	SBS modified asphalt	113	10,576	613.00	1,190,230	68,987
	Aggregate	2355	37	3.50	87,133	8242
Total	\	\	\	\	1,277,364	77,230

For cold central plant recycling and FDR-PC, only cement and water in the base layer materials contribute to carbon emissions. In contrast, the removal and reconstruction also includes aggregates. The carbon emissions and energy consumption of the base layer materials for the three technologies are detailed in Table 6. As shown, the carbon emissions for the removal and reconstruction are approximately 201,000 kg, for the cold central plant recycling approximately 217,000 kg, and for FDR-PC approximately 211,000 kg. The carbon emissions from the raw materials of the two recycling technologies are the highest. This is because, under the same service life of the pavement, the base layer thickness of the recycling technologies is greater, requiring more cement. Given that the carbon emission factor of cement is two to three orders of magnitude higher than that of aggregates and water, the carbon emissions from the raw materials of the recycling technologies are significantly greater.

**Table 6:** Carbon emissions and energy consumption of the base layer in the material production phase for the three technologies.

	Material	Material consumption (t)	Energy consumption per unit (MJ/t)	CO_2_ emissions per unit (kg/t)	Energy consumption (MJ)	CO_2_ emissions (kg)
Removal and reconstruction	Cement	215	3181.00	870	683,192	186,852
	Aggregate	4295	37.00	3.5	158,932	15,034
	Water	215	3.00	0.2	644	43
	Total	/	/	/	842,768	201,929
Cold central plant recycling	Cement	250	3181.00	870	794,390	217,265
	Water	300	3.00	0.2	899	60
	Total	/	/	/	795,289	217,325
FDR-PC	Cement	243	3181.00	870	772,855	211,375
	Water	316	3.00	0.2	948	63
	Total	/	/	/	773,802	211,438

#### Transportation phase

The carbon emissions and energy consumption of the surface layer materials during the transportation phase are presented in Table 7.

**Table 7:** Carbon emissions and energy consumption during the transportation phase of surface layer materials.

Material	Material quantity (t)	Transportation vehicle	Transportation distance (km)	CO_2_ emission factor (kg/t·km)	Energy consumption factor (MJ/t·km)	Total energy consumption (MJ)	Total CO_2_ emission (kg)
SBS Modified Asphalt	113	Dump Truck	40	0.159	4.23	19,042	716
Surface Layer Aggregate	2355	Lorry	20	0.122	3.25	153,072	5746
Surface Layer Asphalt Mixture	2468	Dump Truck	10	0.159	4.23	104,375	3923
Total	\	\	\	\	\	276,489	10,385

The carbon emissions and energy consumption during the transportation phase of base layer materials for the three technologies are detailed in Table 8. The carbon emissions for removal and reconstruction are approximately 25,000 kg, for the cold central plant recycling approximately 16,000 kg, and for the FDR-PC only 1,000 kg, which is an order of magnitude lower than the former two technologies. The reason for this disparity lies in the fact that, during the transportation of base layer materials, both removal and reconstruction and the cold central plant recycling require transporting base layer raw materials to the mixing plant and then transporting the mixed materials from the mixing plant to the construction site. Additionally, removal and reconstruction also necessitates transporting old pavement materials to designated disposal sites. In contrast, FDR-PC only requires transporting a small amount of raw materials directly to the construction site.

**Table 8:** Carbon emissions and energy consumption during the transportation phase of base layer materials.

	Material	Material quantity(t)	Transportation vehicle	Transportation distance (km)	CO_2_ emission factor (kg/t·km)	Energy consumption factor (MJ/t·km)	Total energy consumption (MJ)	Total CO_2_ emission (kg)
Removal and Reconstruction	Cement	215	Lorry	20	0.122	3.25	13,960	524
	Base layer aggregate	4295	Lorry	20	0.122	3.25	279,205	10,481
	Reclaimed material	5775	Lorry	10	0.122	3.25	187,688	7046
	New base layer mixture	4725	Dump Truck	10	0.159	4.23	199,868	7513
	Total	\	\	\	\	\	680,720	25,563
Cold Central Plant Recycling	Cement	250	Lorry	20	0.122	3.25	16,232	609
	Reclaimed material	5775	Lorry	10	0.122	3.25	187,688	7046
	Plant-mixed recycled mixture	5544	Dump truck	10	0.159	4.23	234,511	8815
	Total	\	\	\	\	\	438,431	16,470
FDR-PC	Cement	243	Lorry	20	0.122	3.25	15,792	593
	Water	316	Lorry	10	0.122	3.25	10,265	385
	Total	\	\	\	\	\	26,057	978

### Construction phase

Carbon emissions and energy consumption during the construction phase are presented in Table 9.

**Table 9:** Energy consumption and carbon emissions of surface layers during the construction phase for the three technologies.

	Equipment workload (t)	Energy consumption factor (MJ/t)	CO_2_ emission factor (kg/t)	Total energy consumption (MJ)	Total CO_2_ Emissions (kg)
Asphalt mixture production, paving, and compaction	2468	263.47	10.93	650,112	26,970

The carbon emissions and energy consumption of the base layers during the construction phase for the three technologies are summarized in Table 10. The carbon emissions for removal and reconstruction amount to approximately 22,000 kg, for the cold central plant recycling approximately 24,000 kg, and for FDR-PC approximately 19,000 kg. The marginal differences in carbon emissions among the three technologies can be attributed to the comparable mass of the new pavement mixtures, as well as the shared requirement for operations such as mixing, paving, and compacting of new materials.

**Table 10:** Carbon emissions and energy consumption of base layers during the construction phase for the three technologies.

	Equipment	Equipment workload (t)	Energy consumption factor (MJ/t)	CO_2_ emission factor (kg/t)	Total energy consumption (MJ)	Total CO_2_ emissions (kg)
Removal and reconstruction	Milling machine	5775	34.17	2.53	197,332	14,611
	Inorganic mixture mixing	4725	4.84	0.84	22,869	3969
	Inorganic stabilized mixture paving	4725	7.07	0.27	33,406	1276
	Inorganic stabilized mixture compaction	4725	15.58	0.58	73,616	2741
	Total	/	/	/	327,222	22,596
Cold central plant recycling	Milling machine	5775	34.17	2.53	197,332	14,611
	Plant mixing equipment	5544	4.84	0.84	26,833	4657
	Paver	5544	7.07	0.27	39,196	1497
	Road roller	5544	15.58	0.58	86,376	3216
	Total	/	/	/	349,736	23,980
FDR-PC	Wirtgen cold recycler	5418	38.32	2.62	207,618	14,195
	Grader	5418	4.00	0.25	21,672	1355
	Road roller	5418	15.58	0.58	84,412	3142
	Total	/	/	/	313,702	18,692

### Comparison of three technologies

**Fig. 5:**
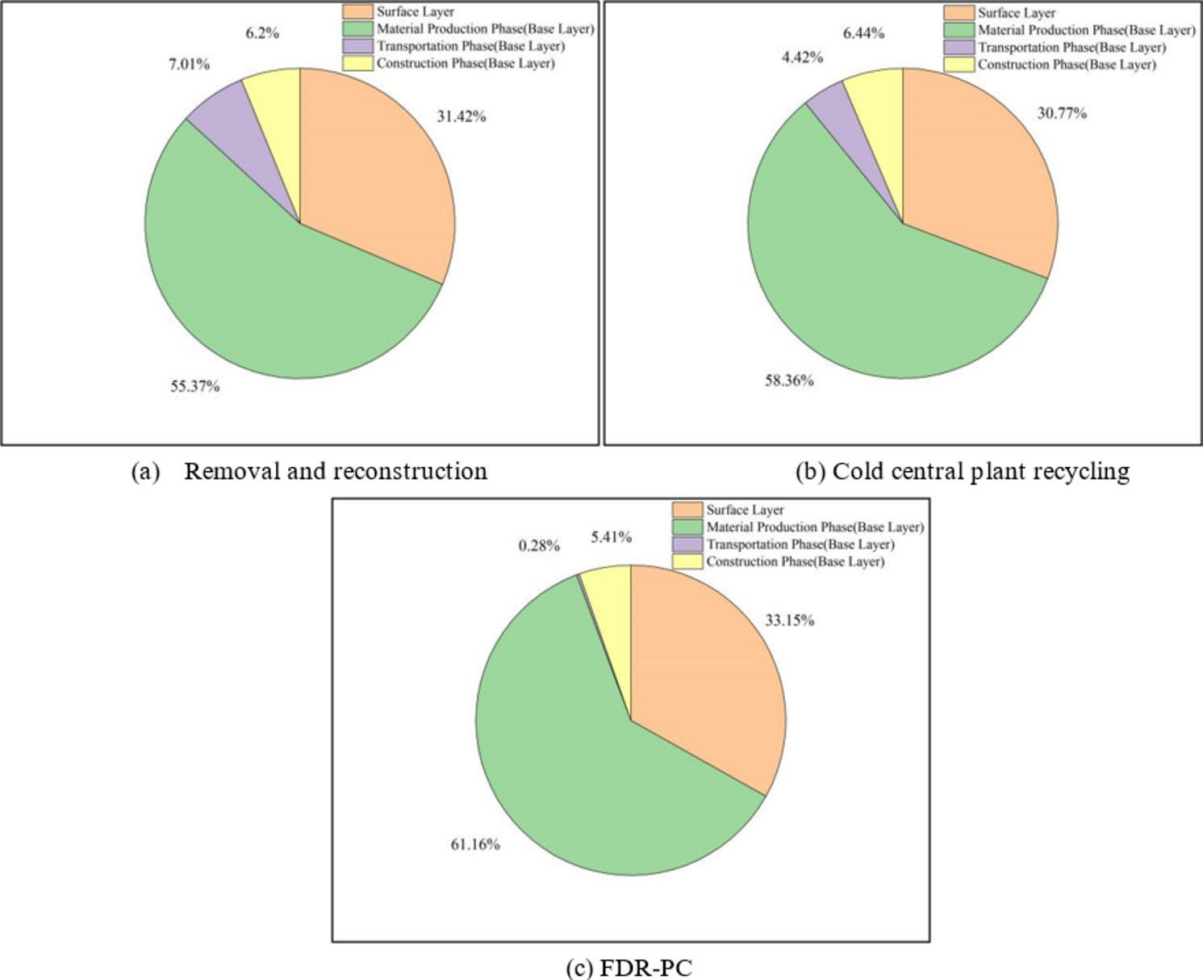
Proportional distribution of CO₂ emissions for three technologies, (**a**) removal and reconstruction, (**b**) cold central plant recycling, (**c**) FDR-PC.

**Fig. 6:**
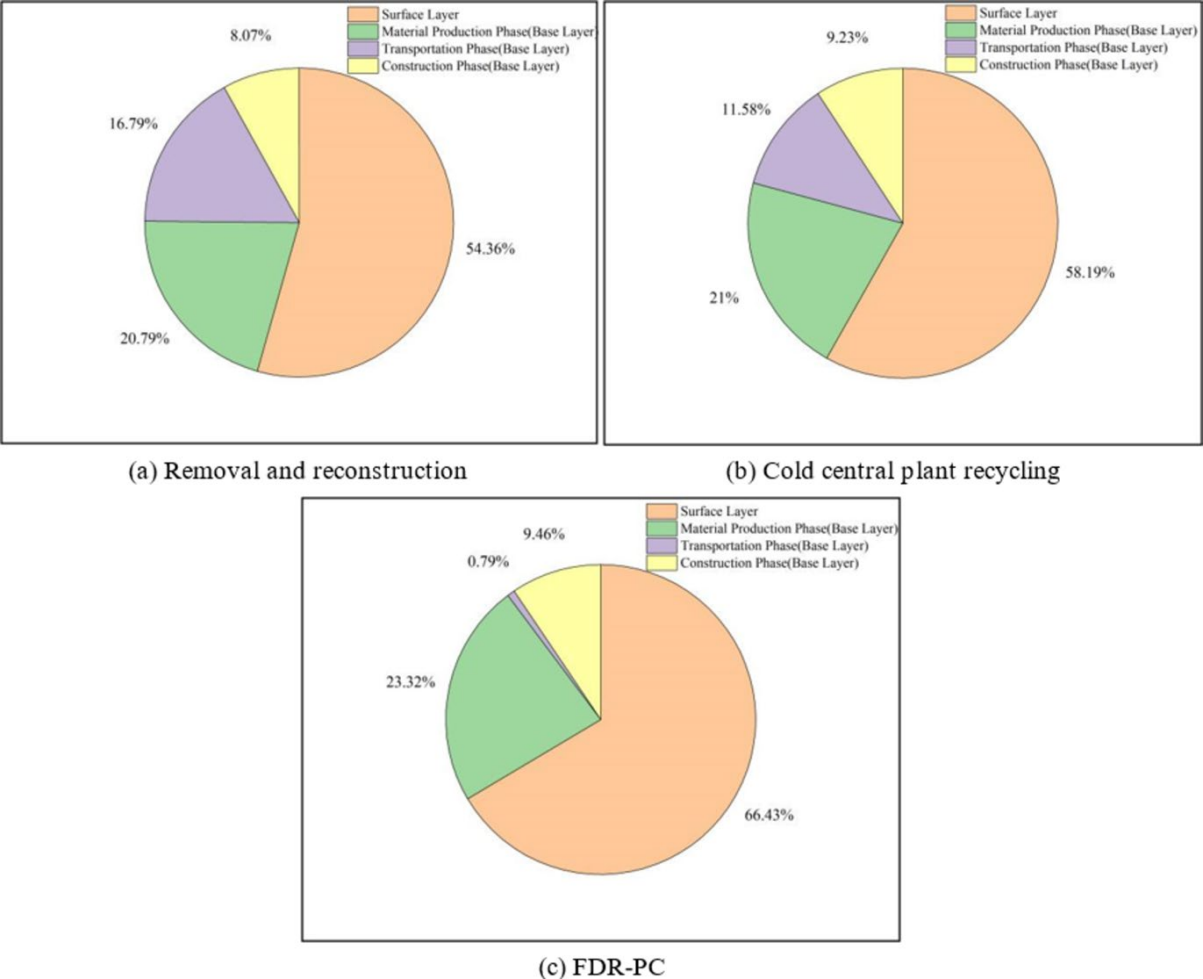
Proportional distribution of energy consumption for three technologies, (**a**) removal and reconstruction, (**b**) cold central plant recycling, (**c**) FDR-PC.

**Fig. 7:**
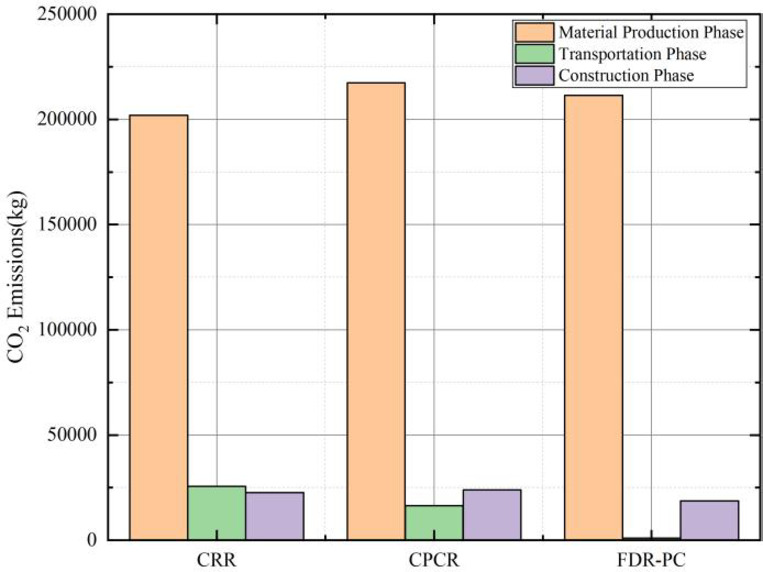
CO₂ emissions across LCA phases for three technologies.

**Fig. 8:**
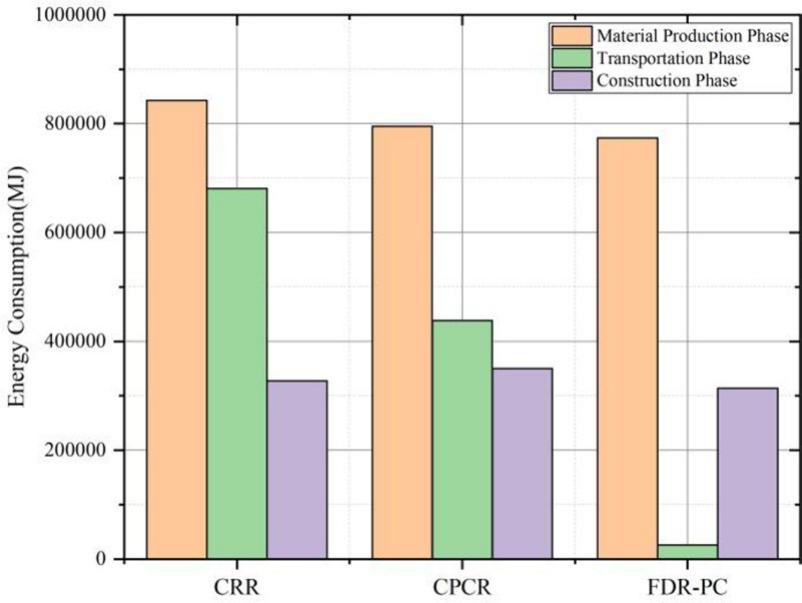
Energy consumption across LCA phases for three technologies.

The aggregated carbon emissions for the three technologies are presented in Table 11, with corresponding visual representations in Figs. 5, 6 and 7. The total energy consumption is detailed in Table 12, illustrated in Figs. 6 and 8. Additionally, Table 13 provides the proportions of base layer carbon emissions and energy consumption for FDR-PC relative to removal and reconstruction and cold central plant recycling.

**Table 11:** Summary of CO_2_ emissions for the three technologies.

	Surface course (kg)	CO_2_ emissions of base course (kg)				Total
		Material production phase	Transportation phase	Construction phase	Total	
Removal and reconstruction	114,585	201,929	25,563	22,596	250,089	364,673
Cold central plant recycling	114,585	217,325	16,470	23,980	257,775	372,359
FDR-PC	114,585	211,438	978	18,692	231,108	345,693

**Table 12:** Summary of energy consumption for the three rehabilitation methods.

	Surface course (kg)	Energy consumption of base course (kg)				Total
		Material production phase	Transportation phase	Construction phase	Total	
Removal and reconstruction	2,203,965	842,768	680,720	327,222	1,850,710	4,054,675
Cold central plant recycling	2,203,965	795,289	438,431	349,736	1,583,457	3,787,422
FDR-PC	2,203,965	773,802	26,057	313,702	1,113,562	3,317,527

**Table 13:** CO₂ emission and energy consumption ratios of FDR-PC relative to removal and reconstruction and cold central plant recycling across LCA phases.

	CO_2_ emissions of the base layer				Energy consumption of the base layer			
	Total	Material production phase	Transportation phase	Construction phase	Total	Material production phase	Transportation phase	Construction phase
Removal and Reconstruction	92%	105%	4%	83%	60%	92%	4%	96%
Cold Central Plant Recycling	90%	97%	6%	78%	70%	97%	6%	90%

In terms of total carbon emissions, the carbon footprint of FDR-PC is 92% of that of removal and reconstruction and 90% of that of cold central plant recycling. Regarding total energy consumption, FDR-PC consumes 60% of the energy used by removal and reconstruction and 70% of that used by cold central plant recycling. FDR-PC exhibits the lowest levels of both carbon emissions and energy consumption, with its energy usage being significantly lower than that of the other two technologies. Structurally, the carbon emissions from the surface layer for each technology are less than those from the base layer, accounting for approximately 30% of the total emissions. Conversely, the energy consumption of the surface layer exceeds that of the base layer, constituting about 60% of the total energy consumption.

An analysis of carbon emissions and energy consumption across various stages of the base layer reveals distinct differences among the three technologies. The carbon emissions and energy consumption during the material production phase are the highest for all three technologies, particularly in terms of carbon emissions, which are substantially higher than those during the transportation and construction phases. A comparison of base layer data indicates that the differences in carbon emissions and energy consumption during the material production phase and construction phases are minimal among the three technologies. The primary advantage of FDR-PC lies in the transportation phase. During transportation, the carbon emissions of FDR-PC technology are 4% and 6% of those of removal and reconstruction and cold central plant recycling, respectively, with energy consumption also at 4% and 6%. FDR-PC utilizes existing road materials on-site to produce new mixtures, thereby eliminating the need to transport old materials and new mixtures. Only a small quantity of cement needs to be transported to the construction site. Given that the mass of the new mixture far exceeds that of the cement, significant reductions in transportation-related emissions and energy consumption are achieved.

In summary, under the same pavement service life, FDR-PC demonstrates the lowest carbon emissions and energy consumption compared to both removal and reconstruction and cold central plant recycling. The environmental benefits of FDR-PC are primarily manifested in the reduction of carbon emissions and energy consumption during the transportation phase. Additionally, the carbon emissions of cold central plant recycling exceed those of removal and reconstruction. This is attributed to the greater thickness of the base layer required by cold central plant recycling under the same pavement service life, which necessitates the use of more cement. Given that cement is a high-carbon emission material, even the recycling of old road materials cannot offset the additional carbon emissions resulting from the increased cement usage.

### Sensitivity analysis

A sensitivity analysis was conducted on the parameters of the carbon emission and energy consumption calculation models for FDR-PC to identify the key factors influencing carbon emissions. The selected parameters for analysis included cement content, water content, transportation distances, the efficiency of lorriy, as well as the efficiency of cold recycling machines, rollers, and graders. The standard values for cement content, water content, transportation distance 1, and transportation distance 2 were set at 5%, 6.5%, 10 km, and 20 km, respectively. Using a step size of 1%, the corresponding carbon emissions and energy consumption were calculated for parameter variations ranging from − 20% to + 20%. For machinery efficiency, the standard value was set at 100%, and the carbon emissions and energy consumption were calculated for parameter variations ranging from − 20 to 0% with a step size of 1%.

**Fig. 9:**
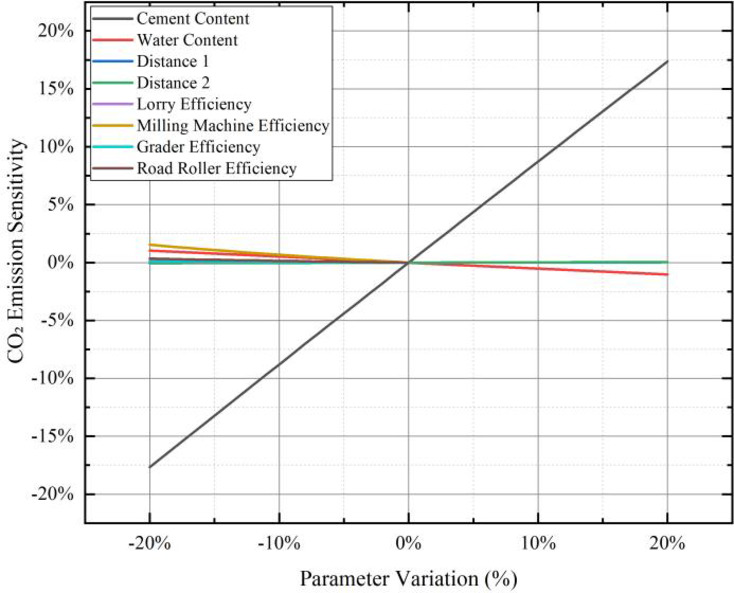
Sensitivity of CO₂ emissions in the FDR-PC carbon emission model.

Figure 9 presents the sensitivity results of the parameters on carbon emissions. It is evident that cement content plays a dominant role in influencing carbon emissions. A 20% change in cement content results in approximately a 17% variation in carbon emissions. In contrast, a 20% change in other parameters leads to a less than 2% variation in total carbon emissions.

**Fig. 10:**
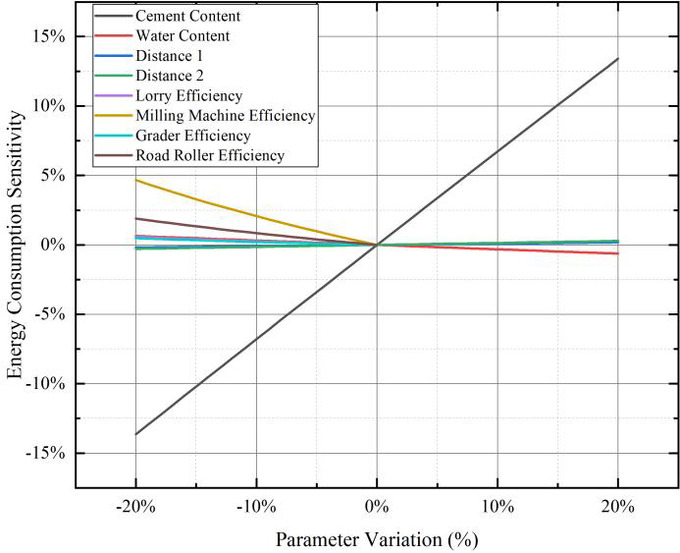
Sensitivity of energy consumption in the FDR-PC energy consumption model.

Figure 10 illustrates the sensitivity results of the parameters on energy consumption. Cement content again exhibits a predominant influence on energy consumption. A 20% change in cement content causes approximately a 12% variation in total energy consumption. For all other parameters, a 20% change results in a less than 2% variation in total energy consumption.

The results of the sensitivity analysis demonstrate that cement content significantly impacts both the carbon emissions and energy consumption outcomes of FDR-PC. The influence of other parameters on carbon emissions is an order of magnitude smaller.

## Conclusion

This study, grounded in the LCA theory, calculates and compares the carbon emissions of three major rehabilitation technologies for ordinary highways under the same pavement service life, with a particular focus on analyzing the sensitivity of the FDR-PC carbon emission model to various carbon emission and energy consumption factors. The conclusions of the study are as follows:

1. Utilizing LCA theory, a carbon emission model was constructed. The results indicate that, under the same pavement service life, the base layer carbon emissions of FDR-PC are 92% and 90% of those of removal and reconstruction and cold central plant recycling, respectively. Similarly, the energy consumption of FDR-PC is 60% and 70% of the aforementioned technologies, respectively. Consequently, FDR-PC demonstrates the most favorable environmental benefits.
2. The carbon emissions and energy consumption of the three technologies during the raw material production and construction phases exhibit minimal differences. However, significant disparities are observed during the transportation phase, where the energy consumption of FDR-PC is nearly negligible.
3. The sensitivity of different parameters in FDR-PC carbon emission and energy consumption calculation models varies significantly. Among these, the sensitivity of cement content to carbon emissions and energy consumption far exceeds that of other parameters.

This study primarily focuses on construction-phase carbon emissions of highway rehabilitation, excluding emissions from the use and maintenance phases. Additionally, the greenhouse gas discussion is limited to CO₂ emissions, while more precise carbon accounting should incorporate CH₄ and N₂O. These aspects will be systematically investigated in subsequent research efforts.

## Data Availability

The datasets used and/or analysed during the current study available from the corresponding author on reasonable request.
